# Group B streptococcal membrane vesicles induce proinflammatory responses in neonatal meninges

**DOI:** 10.1128/iai.00231-26

**Published:** 2026-06-22

**Authors:** Luke R. Joyce, Amanda Brady, Sol Kim, Priya M. Christensen, Kelli L. Palmer, Ziqiang Guan, Julie A. Siegnethaler, Kelly S. Doran

**Affiliations:** 1 Department of Immunology and Microbiology, University of Colorado School of Medicine 12225 , Aurora, Colorado, USA; 2 Department of Pediatrics, Section of Developmental Biology, University of Colorado School of Medicine 12225 , Aurora, Colorado, USA; 3 Department of Biological Sciences, University of Texas at Dallas 12335, Richardson, Texas, USA; 4 Department of Biochemistry, Duke University Medical Center 609772, Durham, North Carolina, USA; University of Illinois Chicago, Chicago, Illinois, USA

**Keywords:** *Streptococcus agalactiae*, membrane vesicle, glycolipids, meninges

## Abstract

*Streptococcus agalactiae* (Group B *Streptococcus*; GBS) is the leading cause of neonatal meningitis, resulting in morbidity and lasting neurological effects in survivors. GBS makes three unique glycolipids in their cell membrane, which we have previously shown to promote bloodstream survival and meningitis. GBS also produces membrane vesicles (MVs), but the lipid content is not known, nor is it known how glycolipids impact MV biogenesis, cargo, and function. Additionally, it is currently unknown how GBS MVs may contribute to systemic infection, meningitis, and resulting neuroinflammation. Here, using our isogenic lipid mutants in a clinically relevant sequence type 17 and capsule type III strain, we identify that the MV lipidome replicates that of the parent bacterial cell, and that MVs isolated from a glycolipid-devoid strain have an altered MV proteome. Investigating the impact of GBS MVs on brain endothelial cells *in vitro,* we observe significant increases in proinflammatory chemokines CXCL-1 and IL-6 from MVs isolated from WT and lipid mutant strains. Furthermore, we show that the proteins on the MV surface contribute to cytokine induction, which is dependent on Toll-like receptor 2 (TLR2)-mediated signaling. Finally, using a neonatal murine infection model, we observe significant increases in proinflammatory chemokines CXCL-1, IL-6, TNFα, and IL-1β in the murine leptomeninges and brain when GBS MVs are present in the cerebrospinal fluid. This work reveals unexplored functions of bacterial glycolipids in MV biogenesis and identifies GBS MVs as potent inflammatory molecules to the meninges and brain endothelium that may contribute significantly to meningitis disease progression.

## INTRODUCTION

*Streptococcus agalactiae* (Group B *Streptococcus*; GBS) is a gram-positive bacterium that frequently colonizes the gastrointestinal tract and female reproductive tract asymptomatically (1–3); however, it is also the leading cause of neonatal meningitis, with mortality rates of up to 9% (4) and neurological sequelae in survivors (5–8). There are multiple phylogenetic sequence types (ST) within GBS; however, ST-17, which is predominantly comprised of capsule type III, is highly associated with neonatal meningitis (9–11). For GBS to cause meningitis, it must spread systemically through the bloodstream, interacting with immune cells and the cerebrovasculature of the central nervous system (CNS), such as the blood-brain barrier (BBB) and the blood-cerebrospinal fluid (CSF) barrier of the leptomeninges (2, 12). Thus, GBS has a variety of virulence factors that contribute to systemic spread and inflammation, resulting in disruption of the CNS barriers and the development of meningitis (2, 12). Previously, we investigated the biosynthetic pathways for three glycolipids present in the GBS cell membrane and observed the importance of the glycolipids during systemic infection and interactions with the cerebrovasculature (13–15). The enzyme IagB synthesizes the first glycolipid, glucosyl-diacylglycerol (Glc-DAG), and GBS∆*iagB* is devoid of all three glycolipids, resulting in GBS being highly susceptible to neutrophil killing (13). Enzyme IagA adds a second glucose to Glc-DAG to form Glc_2_-DAG, the well-known lipoteichoic acid (LTA) lipid anchor (15). The enzyme MprF adds a lysine amino acid onto Glc-DAG to form Lysyl-Glc-DAG, an amino-acylated glycolipid; MprF also synthesizes Lysyl-phosphatidylglycerol (Lys-PG) (14). Interestingly, we observed that both GBS∆*iagA*, lacking Glc_2_-DAG, and GBS∆*mprF,* lacking Lys-Glc-DAG and Lys-PG, survive in the murine bloodstream but are important for penetrating the brain and causing meningitis (14, 15).

Membrane vesicles (MVs) are produced by almost all gram-positive bacteria and are spherical, bilayered nanostructures that transport a diverse repertoire of cargo, including proteins, lipids, and RNA that interact with host cells (16, 17). Within the streptococci, MVs from *Streptococcus pneumoniae* have been shown to modulate opsonophagocytic uptake by macrophages and alter macrophage cell signaling (18, 19), and MVs from *S. mitis* cause inflammation in epithelial cells, neutrophils, and macrophages (20). Recent studies have demonstrated that GBS MVs are packaged with virulence factors, including host matrix-degrading enzymes such as HylB and the GBS β-hemolytic cytolysin/pigment, and when GBS are treated with antibiotics, alterations in MV production and cargo are observed (21–25). GBS MVs injected into the murine amniotic sac cause chorioamnionitis-like inflammation, resulting in preterm birth (21), and when incubated with macrophages *in vitro,* they induce the NLRP3 inflammasome (22). While these previous studies have begun to elucidate the mechanisms of GBS MV-mediated inflammation, it is unknown how GBS MVs impact the cerebrovasculature and aid in meningitis progression in neonates.

The membranes of GBS MVs have been observed to contain a similar fatty acid composition to that of the cellular membrane (21), which has also been observed in *S. pyogenes* (26). However, the phospholipid and glycolipid composition of GBS MVs is unknown. We have previously characterized the importance of GBS glycolipids and amino-acylated lipids during meningitis disease progression (13–15) and in the diabetic wound (27); however, it is still unknown how these parent cell-lipid alterations impact MV biogenesis, cargo packaging, and host cell interactions. In this study, we utilized a series of isogenic lipid mutants that lack glycolipids and/or lysine-modified lipids to determine how these modifications affect MV biogenesis and cargo. We characterized the proinflammatory potential of GBS MVs in human brain microvascular endothelial cells *in vitro* and in the leptomeninges and brain of neonatal mice *in vivo*. Furthermore, we observed that inflammation in brain endothelial cells is dependent on the surface proteins of MVs and is Toll-like receptor 2 (TLR2)-mediated. Our findings provide new insights into MV biogenesis and how GBS utilizes MVs to induce inflammation and promote pathogenesis.

## RESULTS

### Phenotypic characterization of MVs derived from COH1 and glycolipid mutants

To investigate the potential impact of the parent cell lipid composition on GBS MVs, we utilized the commonly used GBS ST-17 and capsule type III strain, COH1, a clinical isolate from a neonatal infection (28). GBS ST-17 strains are predominantly comprised of capsule type III and are the leading cause of both early- and late-onset neonatal disease (9, 10); thus, characterizing and profiling MVs produced by these strains is of clinical significance. It is known that GBS MV membranes have fatty acid compositions similar to that of the GBS whole cell (21); however, it is unknown what lipid head groups are present. To identify the lipid composition of COH1 WT MVs (MV-COH1), we performed comprehensive lipidomics on MV-COH1 and confirmed the presence of phospholipids: phosphatidylglycerol (PG), Lysyl-PG (Lys-PG), cardiolipin (CL), and GBS’s three glycolipids: glucosyl-diacylglycerol (Glc-DAG), diglucosyl-diacylglycerol (Glc_2_-DAG), and lysyl-glucosyl-diacylglycerol (Lys-Glc-DAG) (Table 1); thus, GBS MV lipidomes replicate the composition of the bacterial cell membrane (13, 14, 29).

**TABLE 1:** Lipids present in GBS membrane vesicles

	PG	CL	Glc-DAG	Glc_2_-DAG	Lys-PG	Lys-Glc-DAG
MV-COH1^*a*^	**+**	**+**	**+**	**+**	**+**	**+**
MV-∆*iagB*^*b*^	**+**	**+**	**-**	**-**	**+**	**-**
MV-∆*iagA*^b^	**+**	**+**	**+**	**-**	**+**	**+**
MV-∆*mprF*^b^	**+**	**+**	**+**	**+**	**-**	**-**

^*a*^ Lipids are detected by LC-MS/MS in MV-COH1 and present in GBS parent cells (13, 14, 29).

^*b*^ Lipid profiles of GBS lipid mutants are previously published (13–15).

**Fig 1:**
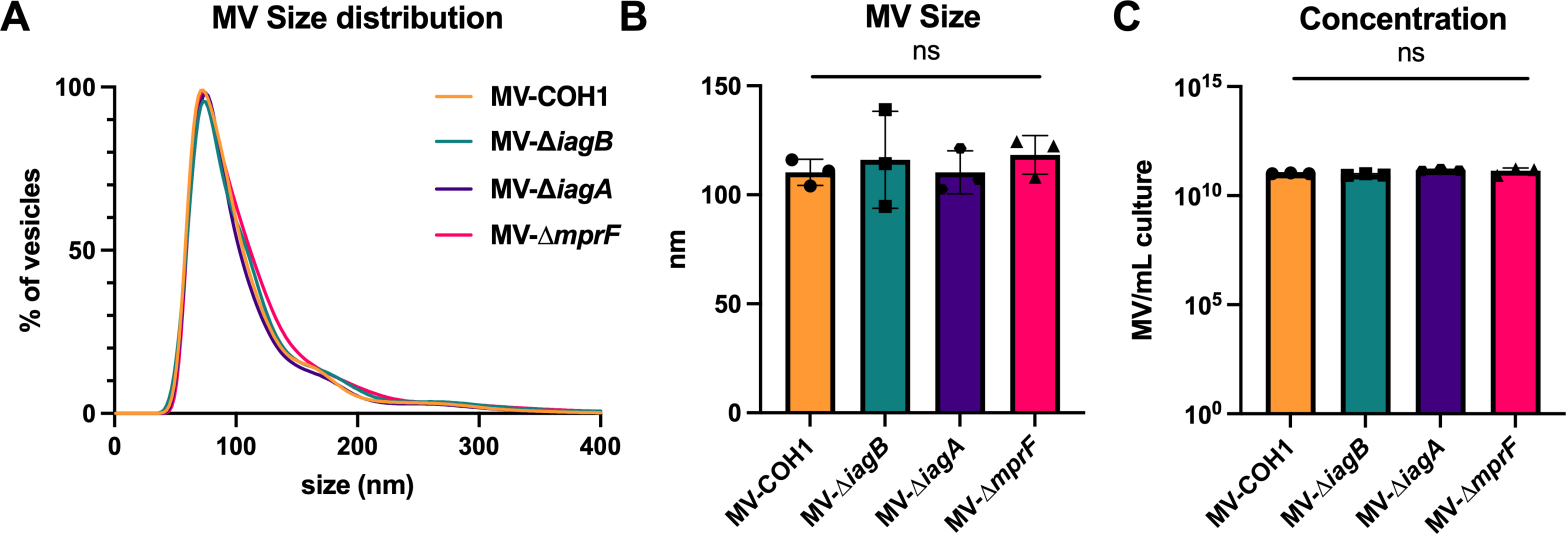
Characterization of membrane vesicles derived from COH1 WT and lipid mutants. (**A**) Size distribution as percentage of total MV, (**B**) average size of MVs, and (**C**) number of MVs produced per 1 mL of bacterial culture. No significant differences are observed between MVs derived from WT or lipid mutants. (**B** and **C**) Mean and SD indicated. (**B** and **C**) Statistical analyses were performed using one-way ANOVA with Fisher’s LSD test. ns; not significant.

It is currently unknown how the lipid composition of the bacterial cell will impact GBS MV formation and characteristics. We next sought to assess this by utilizing our isogenic lipid mutants: COH1∆*iagB,* which lacks all glycolipids (MV-∆*iagB*) (13), COH1∆*iagA* lacking Glc_2_-DAG (MV-∆*iagA*) (15), and COH1∆*mprF,* which lacks the lysine lipids Lys-PG and Lys-Glc-DAG (MV-∆*mprF*) (14) (Table 1). MVs isolated from each strain underwent nanoparticle-tracking analysis to assess the size and quantity of MVs produced by each strain when grown under regular laboratory conditions. No significant differences were observed in the size distribution, average size, and the concentration of MVs produced by each strain when compared to MV-COH1 (Fig. 1A through C), indicating that the cell lipid composition does not impact the biogenesis of GBS MVs under normal laboratory conditions.

### Parent cell lipid composition impacts MV proteome

**Fig 2:**
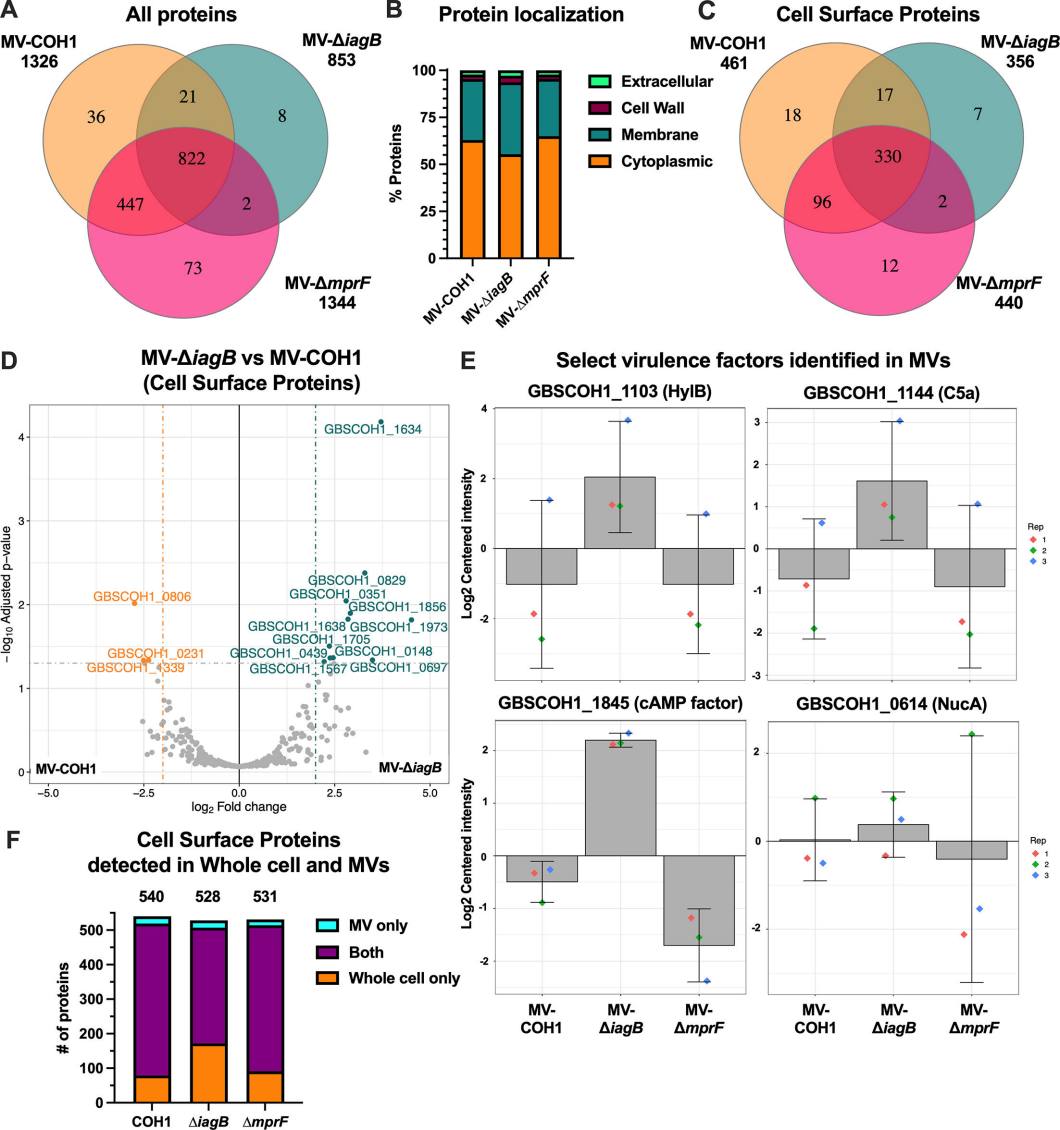
GBS glycolipids alter the MV proteome. LC-MS/MS identification of proteins in MV-COH1, MV-∆*iagB,* and MV-∆*mprF*. (**A**) Total number of proteins and (**B**) the proportion of proteins detected based on cell localization, as determined by pSortDB and Cello, indicate that while MV-∆*iagB* has fewer total proteins detected, more cell surface proteins are present compared to the other MVs. (**C**) Total number of cell surface proteins identified in each MV, and (**D**) differential abundance of cell surface proteins in MV-∆*iagB* compared to MV-COH1. MV-∆*iagB* has increased abundance of more proteins compared to MV-COH1. (**E**) Known GBS virulence factor abundance detected in MVs, 95% confidence interval. (**F**) The total number of cell surface proteins detected in live stationary phase GBS and their associated MVs, and numbers above indicate the total number of cell surface proteins detected in live GBS. Proteomics is performed in biological triplicate.

Proteins are a major cargo harbored by GBS MVs and are variable between differing serotypes (23). We next assessed the impact of glycolipids and the lysine-modified lipids on the MV proteome using nano-LC-MS/MS and analysis via DEP2 proteomic analysis software (30). We detected a total of 853 proteins in MV-∆*iagB*, far fewer proteins than in both MV-COH1 (1,326) and MV-∆*mprF* (1,344) (Fig. 2A and Table S1). MVs from each strain background had a set of unique and shared proteins, with a core set of 822 proteins common between all the MVs. A principal component analysis indicated the protein composition of each MV is dependent on the lipid profile (Fig. S1A), and a hierarchical clustering of all proteins indicated that the proteomic profile between MV-COH1 and MV-∆*mprF* is more similar compared to that of MV-∆*iagB* (Fig. S1B). Additionally, identifying the subcellular localization of proteins revealed a few differences between the MVs; however, MV-∆*iagB* had fewer cytoplasmic proteins and more cell membrane proteins as a proportion of its total proteins compared to the MVs isolated from the WT and ∆*mprF* strains (Fig. 2B).

To further assess the proteomes of these MVs, we focused on the cell surface-localized proteins by combining proteins identified as cell membrane and cell wall, since these proteins may contribute to initial interactions with the host. MV-COH1 had the most cell surface proteins detected at 461, with MV-∆*mprF* containing 440, and MV-∆*iagB* with 356 (Fig. 2C and Table S1). Differential abundance of shared cell surface proteins was performed between MV-COH1 and MV-∆*iagB* or MV-∆*mprF* using DEP2. Interestingly, MV-∆*iagB* had 11 significantly increased protein abundances (padj <0.05; Log2Fold change >2), and only three significantly decreased abundances (padj <0.05; Log2Fold change <-2) (Fig. 2D and Table S2) compared to MV-COH1, whereas MV-∆*mprF* had no significantly altered proteins (Fig. S1C and Table S3). The most significantly increased protein in MV-∆*iagB* is DltD compared to MV-COH1 (GBSCOH1_1634), an enzyme involved in adding alanine modifications to LTA, and the most Log2FoldChange increased protein, GBSCOH1_1973, is a highly conserved surface-associated protein, which has a high antigenic potential and may be a candidate for GBS vaccine development (31). Previous studies have identified known GBS virulence factors associated with MVs (21, 23). We also detect factors such as HylB, the hyaluronate hydrolase, C5a peptidase, cAMP factor, and the nuclease NucA (Fig. 2E). Interestingly, Log2-centered intensity of the proteins to assess the relative abundance between the MVs indicates that HylB, C5a peptidase, and cAMP factor are increased in abundance in MV-∆*iagB* compared to MV-COH1 and MV-∆*mprF*. Taken together, these data indicate that GBS glycolipids play an important role in the MV proteome. While the loss of glycolipids in the bacterial cell results in the decrease of the total number of proteins identified in MVs, the abundance of these proteins is increased compared to WT MVs and may have implications for interactions with the host.

### MV cell surface proteome reflects that of the GBS bacterial cell

We next assessed how the GBS cell surface proteome is reflected in MVs. We isolated cell surface proteins from COH1, ∆*iagB*, and ∆*mprF* after ~18 h of growth, similar to when MVs are collected, and performed comprehensive LC-MS/MS identification (Table S4). A similar number of total proteins were identified between all three strains, with ∆*iagB* possessing the fewest number of proteins (Fig. S2A), and a principal component analysis revealed no overlap between cell surface proteomes (Fig. S2B). Interestingly, differential abundance analysis of proteins present in live cells from ∆*iagB* (Fig. S2C) and ∆*mprF* (Fig. S2D) compared to COH1 revealed few alterations; ∆*iagB* had 14 significantly decreased proteins (padj <0.05; Log2Fold change <-2) and only one significantly increased protein (padj <0.05; Log2Fold change >2), while ∆*mprF* had 10 significantly decreased proteins and six significantly increased proteins (Table S5). This is converse of the MV proteome described above, where MV-∆*iagB* is observed to have more significantly increased proteins compared to MV-COH1. Finally, we compared the cell surface proteomes of the parent cell and respective MVs, and a principal component analysis revealed that the bacterial whole cell proteomes clustered tightly together; however, the MV proteomes were more dispersed and variable (Fig. S2E). We also observed that WT COH1 and ∆*mprF* strains are most similar in the total number of proteins detected in the parent cell and MVs, whereas MV-∆*iagB* has fewer cell surface proteins identified than in the bacterial cell (Fig. 2F). These proteomic data suggest that while the cell surface proteome of MVs replicates that of the bacterial cell membrane, the loss of GBS glycolipids reduces the number of cell surface proteins that are packaged into MVs.

### Lipid mutant-derived MVs have variable RNA cargo

MVs are well known to contain RNA that may shape host cell interactions (16, 32). To assess the impact of an altered lipid membrane composition on RNA content of the MV preparations, we performed RNA sequencing of MV-COH1, MV-∆*iagB,* and MV-∆*mprF*. A principal component analysis of gene-coding transcripts identified little overlap between the different MVs, with each MV clustering closely with itself; however, the MV-∆*iagB* had the largest variability within its gene-coding transcripts compared to the other MVs (Fig. S3A). Of the total number of gene-coding transcripts present in each MV, MV-COH1 had the largest number of transcripts at 119 compared to MV-∆*iagB* with 51 and just 15 for MV-∆*mprF* (Fig. S3B). Each MV possessed unique transcripts with 80 in MV-COH1, 15 in MV-∆*iagB*, and 6 in MV-∆*mprF*. Furthermore, of the transcripts identified in MV-∆*iagB,* 18 were significantly overrepresented (padj <0.05; Log2Fold change >2) compared to MV-COH1 with only one significantly underrepresented (padj <0.05; Log2Fold change <−2), whereas MV-∆*mprF* had eight significantly overrepresented transcripts (Fig. S3C). These data suggest that the lipid composition alters what gene-coding transcripts are packaged into MVs, which may impact host cell interactions.

### MVs induce cytokine production in brain endothelial cells

**Fig 3:**
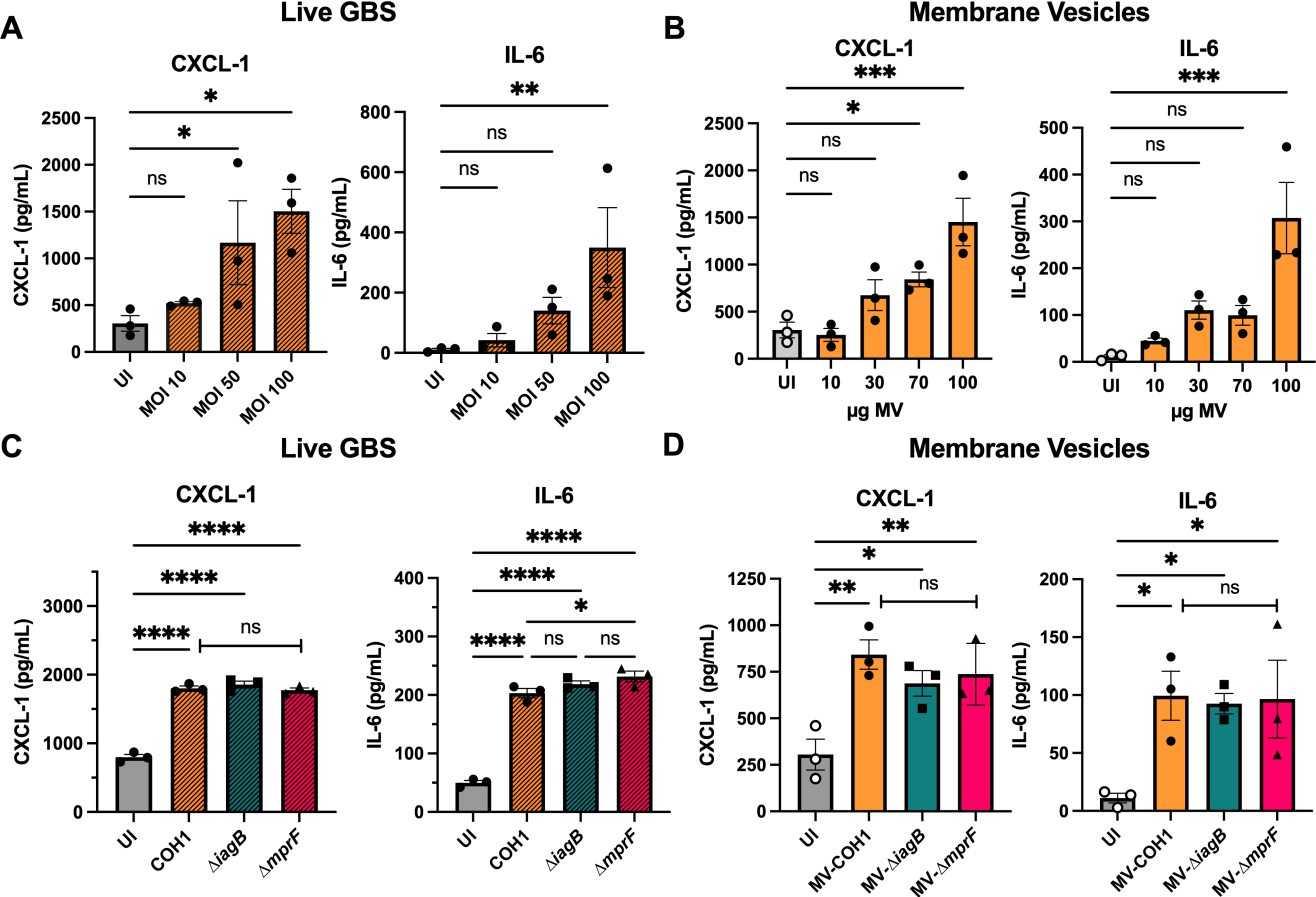
Membrane vesicles induce inflammation in brain endothelial cells *in vitro*. *In vitro* hCMEC monolayer inflammatory signaling (CXCL-1 and IL-6) measured by ELISA when infected with live GBS and MVs. (**A**) Increasing MOI of live GBS infection and (**B**) dose-dependent MV-COH1 infection indicate that both live GBS and MVs induce inflammatory signaling in a dose-dependent manner in hCMECs. CXCL-1 and IL-6 induction at (**C**) an MOI of ~50 for live GBS strains or (**D**) 70 µg of MVs. Both CXCL-1 and IL-6 are significantly increased when live GBS strains or MVs are present compared to uninfected controls. Mean and SEM are indicated. (**A through D**) Statistical analyses were performed using one-way ANOVA with Fisher’s LSD test. *P < 0.05, **P < 0.01, ***P < 0.001, ****P < 0.0001. ns; not significant.

GBS MVs are known to cause inflammation in choriodecidua tissue (21) and macrophages (22); however, it is unknown how MVs impact brain endothelial cells (bECs). We investigated the ability of live COH1 and MV-COH1 to induce cytokine release in bECs using human cerebral microvascular endothelial cells (hCMECs) *in vitro*. After incubating COH1 at varying multiplicity of infection (MOI) and MV-COH1 at varying protein concentrations with hCMECs for 5 h, we observed a significant increase in proinflammatory chemokine CXCL-1 and cytokine IL-6 in a dose-dependent manner (Fig. 3A and B). As we observed differences in the MV proteomes above, we next assessed the proinflammatory response of hCMECs to live COH1, COH1∆*iagB,* and COH1∆*mprF* strains at an MOI of ~50 or 70 µg of MV-COH1, MV-∆*iagB*, and MV-∆*mprF* for 5 h (Fig. 3C and D). Live GBS strains and MVs elicited a significant increase in both CXCL-1 and IL-6, compared to uninfected controls, and live GBS induced higher levels of CXCL-1 and IL-6 compared to MVs. Interestingly, no significant difference was observed between GBS strains or MVs isolated from WT and mutant GBS strains, except for COH1∆*mprF* inducing significantly higher IL-6 compared to COH1 WT (Fig. 3C). To assess if MV treatments caused cytotoxicity, we incubated hCMECs with 70 µg of MVs from WT and mutant strains for 24 h and performed a lactate dehydrogenase (LDH) release assay (Fig. S4A). MVs, regardless of the parent strain isolated from, induced a low amount (~3%) of LDH release compared to untreated controls. These data indicate that bECs induce proinflammatory cytokines in a dose-dependent manner in response to GBS MVs and that the MV lipid composition and the altered proteome do not impact the production of these cytokines.

### Cytokine production is induced by MV surface proteins and dependent on TLR2 signaling

**Fig 4:**
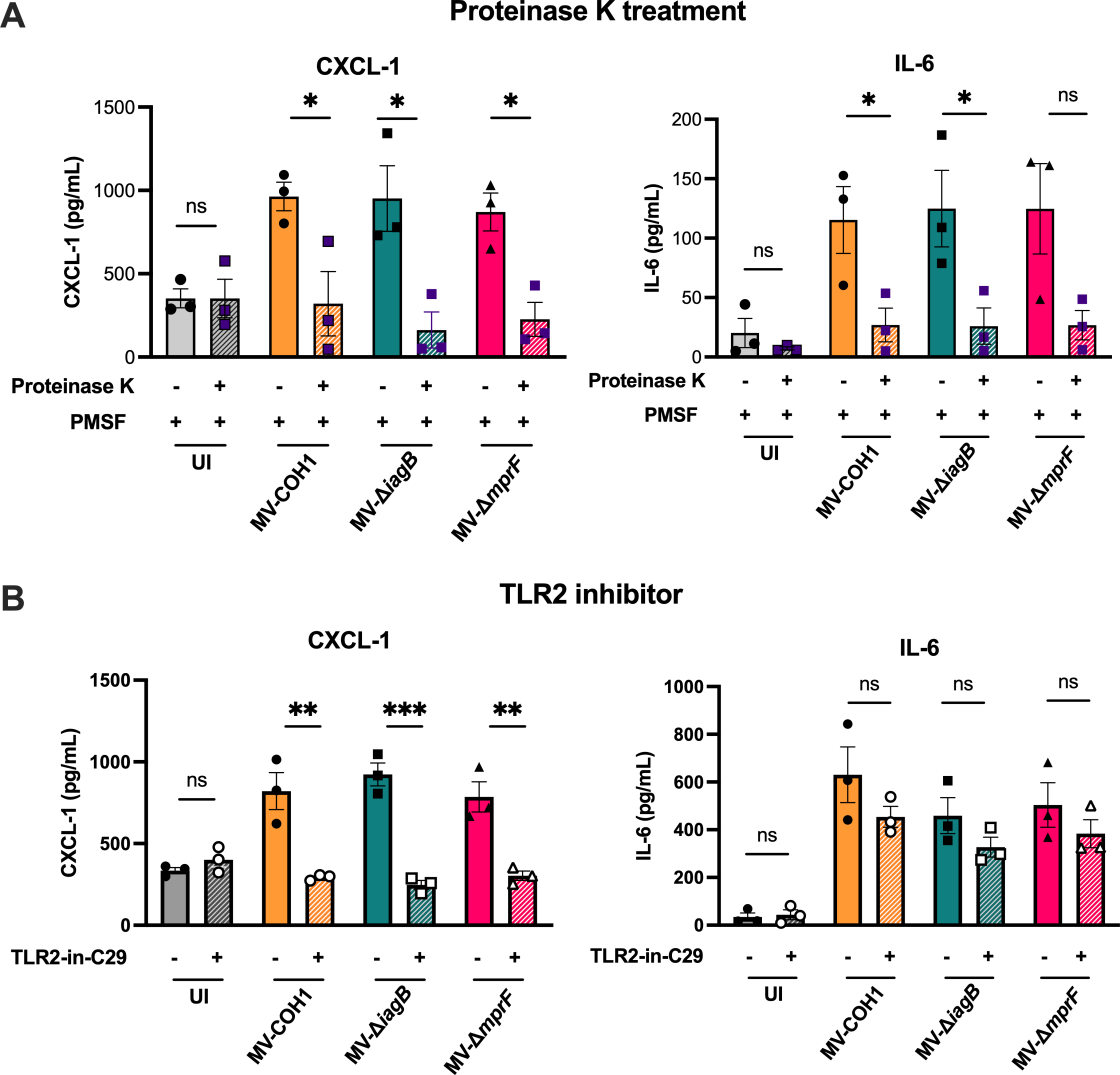
Proteinase treatment and TLR2 inhibition reduce inflammation. *In vitro* inflammation of hCMEC monolayer infected by either (**A**) MVs pretreated with proteinase K or (**B**) TLR2 inhibitor and vehicle control measured by ELISA. A significant decrease is observed when MVs are pretreated with proteinase K and when hCMECs are treated with a TLR2 inhibitor. Mean and SEM are indicated. (**A and B**) Statistical analyses were performed using unpaired two-tailed *t*-test between untreated and proteinase K or TLR2 treated MVs, *P < 0.05, **P < 0.01, ***P < 0.001. ns; not significant.

We also assessed the impact of both the MV proteome and the role of TLR2 signaling, which is known to play an important role in detecting bacterial pathogens, on the bECs response to GBS MVs. We pretreated MVs with proteinase K to degrade surface-associated proteins (Fig. S4B) to see if this altered cytokine induction. Interestingly, proteinase K treatment of MVs resulted in a significant decrease and return to the baseline levels of both CXCL-1 and IL-6 compared to untreated MVs (Fig. 4A). Inhibition of TLR2 using the small molecule inhibitor, TLR2-in-C29, significantly decreased the levels of CXCL-1 compared to vehicle control, as well as a reduction in the levels of IL-6 (Fig. 4B). Altogether, these data suggest that the presence of GBS proteins in MVs is a major driver of inflammation in bECs and that TLR2 is playing a role in the induction of CXCL-1 in response to the MVs to trigger neutrophil recruitment to the cerebrovasculature.

### GBS MVs induce inflammation in murine leptomeninges and brain

**Fig 5:**
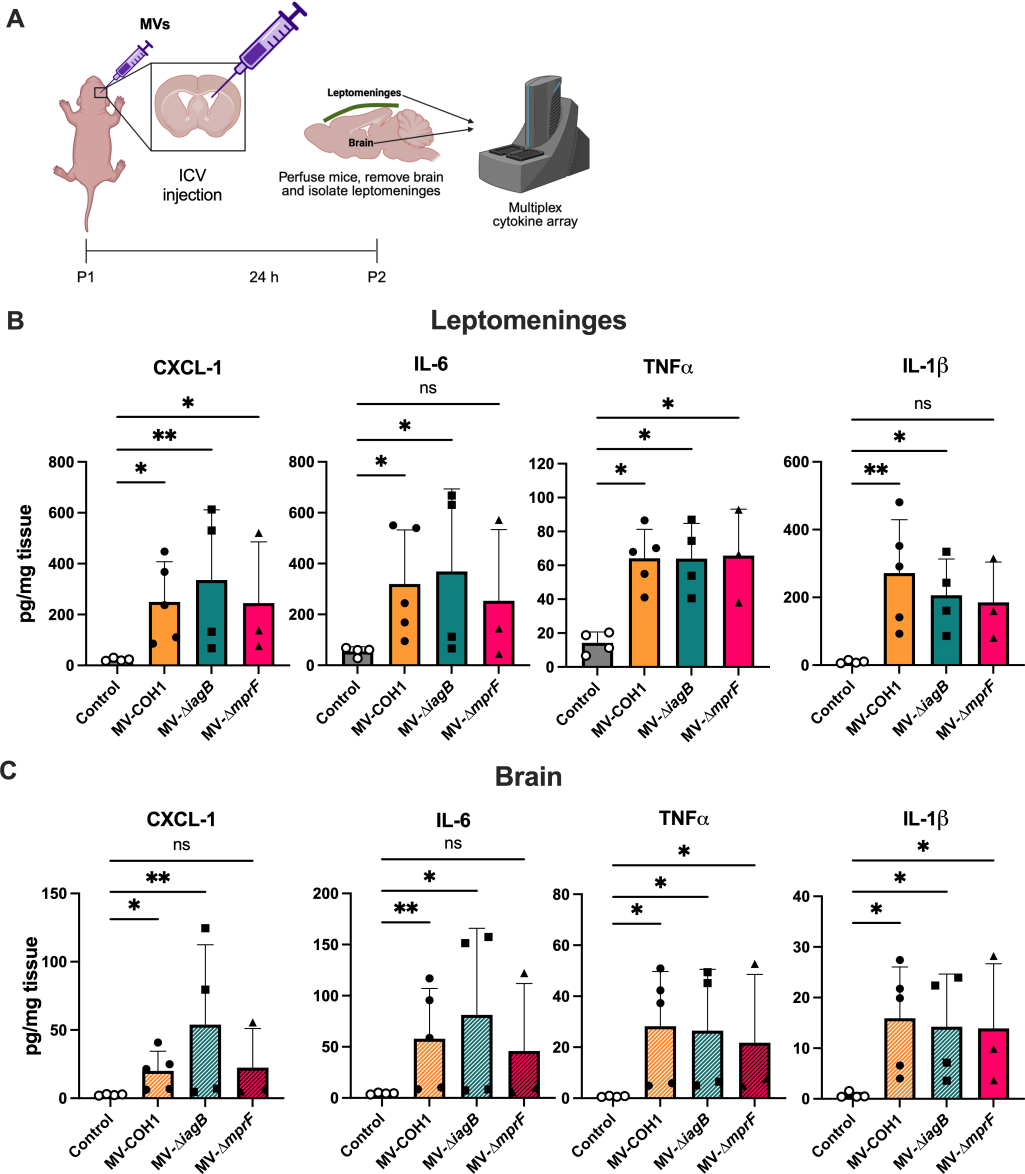
Membrane vesicles induce inflammation in murine leptomeninges and brain tissue. (**A**) Schematic of intracerebroventricular injection (ICV) of MVs into postnatal day 1 (P1) pups and isolation of the leptomeninges and brain tissue after 24 h. Multiplex quantitation of CXCL-1, IL-6, TNFα, and IL-1β in the (**B**) leptomeninges and (**C**) brain tissue. MVs induce a significant increase in inflammation signaling in both the leptomeninges and brain tissue of mice compared to control liposomes. Control: *n =* 4; MV-COH1: *n =* 5; MV-∆*iagB: n* = 4; and MV-∆*mprF: n* = 3. Mean and standard deviation are indicated. (**B and C**) Statistical analyses were performed using Kruskal-Wallis with uncorrected Dunn’s test. *P < 0.05. **P < 0.01, ns; not significant.

Finally, to further assess if GBS MVs induce inflammation in the leptomeninges and brain tissue *in vivo*, we utilized an intracerebroventricular (ICV) neonatal infection model (33). Postnatal day 1 (P1) mice were ICV injected with 9 µg of MV-COH1, MV-∆*iagB*, and MV-∆*mprF* to allow for the MVs to spread via the CSF in the subarachnoid space of the leptomeninges (34). Mice were monitored for 24 h before collecting the leptomeninges and brain tissue for multiplex cytokine analysis (Fig. 5A). While we observed variability between MV treated mice, a significant increase in CXCL-1, IL-6, TNFα, and IL-1β was detected in response to MV-COH1, MV-∆*iagB*, and MV-∆*mprF* compared to liposome controls in both the leptomeninges and brain tissues of mice (Fig. 5B and C). We have previously observed significant increases in these same proinflammatory cytokines, in the same tissues, in response to live GBS challenge (34). Interestingly, the level of inflammatory signaling in the leptomeninges was observed to be much higher compared to the brain, and we observed no significant differences between MV treated mice, similar to our *in vitro* observations. Together, these data suggest that GBS MVs are sufficient to initiate inflammatory signaling in the leptomeninges, which may exacerbate GBS meningitis progression by increasing the level of inflammation.

## DISCUSSION

In this study, we demonstrate for the first time that GBS MVs are potent mediators of proinflammatory responses in the cells of the cerebrovasculature and the meninges and that their cargo is significantly influenced by the lipid composition of the bacterial membrane. We characterized the lipidomic profile of MVs derived from GBS strain COH1, a ST-17 and capsule type III clinical isolate, and found that they largely mirror the lipid composition of the bacterial cell membrane. This is consistent with the current understanding of MV biogenesis in gram-positive bacteria, where vesicles bud from the cell membrane before traversing the thick peptidoglycan cell wall (35).

Previous studies have observed that GBS strains of varying sequence and capsule types possess unique proteomes (23). To assess the impact of the cell membrane lipid composition on MVs within the same sequence and capsule type, we isolated MVs from our isogeneic glycolipid mutants and observed that the loss of all three glycolipids (MV-∆*iagB*) and the two lysine lipids (MV-∆*mprF*) did not alter the size and the quantity of MVs produced in each strain, although the impact of CFU on MV production warrants further investigation. However, we did observe that the variety and number of RNA detected in each MV was altered and that the proteome was significantly altered by the change in lipid composition under routine laboratory culturing conditions. When *Staphylococcus aureus* is grown under different temperatures, alterations in both the transcripts and proteins packaged into MVs are observed, and these changes impact the cytotoxicity of staphylococcal MVs (32). Additionally, when *S. aureus* and GBS are exposed to antibiotics, MV production and cargo are altered (25, 36). Changes to MV cargo based on the conditions the parent cells are in, whether induced by temperature, media, or stressors like antibiotics, can lead to impactful changes to the MV cargo that can determine how the host cell responds to their presence.

Proteins present in MVs are crucial for interactions with host cells (37–39); however, it was not known how the bacterial cell lipid composition would affect MV protein packaging. We observed that MV-∆*iagB* had significantly fewer proteins identified, 853 compared to 1,326 and 1,344 identified in MV-COH1 and MV-∆*mprF*, respectively. Interestingly, no significant abundance differences were observed between cell surface proteins of MV-COH1 and MV-∆*mprF,* but MV-∆*iagB* had 11 proteins significantly increased in abundance compared to just three significantly decreased in abundance. These alterations suggest that GBS glycolipids play a more impactful role in protein packaging, whether through protein localization or abundance in the parent cell. To investigate this, we isolated and identified the cell surface proteins from parent cells. While we observed a similar number of proteins between different strains, clearly, the MV-∆*iagB* contained fewer cell surface proteins than its parent cell, further confirming the importance of glycolipids to MV protein packaging. Alterations to the bacterial lipid membrane, like the loss of the glycolipids, may impact protein localization, anchoring, and secretion (17) and could be a reason why there is altered protein packaging in the MVs, although further investigation is needed to identify exact mechanisms.

Despite the alterations in MV proteomes observed, all GBS MVs retained the ability to induce robust proinflammatory responses of human bECs *in vitro*. MVs induced the production of CXCL-1 and IL-6 in a dose-dependent manner, with the MV-∆*iagB* and MV-∆*mprF* mutants exhibiting similar inflammatory signaling to MV-COH1. A possible explanation is that some of the known GBS virulence factors such as HylB, cAMP factor, and the C5a peptidase were generally more abundant in MV-∆*iagB* compared to MV-COH1 and MV-∆*mprF*. The most significantly increased proteins in MV-∆*iagB* are DltD, involved in adding LTA decoration; GBSCOH1_1973, a highly conserved surface-associated protein with a high antigenic potential (31); and a putative fibrinogen binding protein, GBSCOH1_0697. The inflammatory potential of these proteins is unknown, but these data suggest that they may contribute to inflammation. The combination of known virulence factors packaged into the MVs and the inflammatory nature of the surface proteome of GBS could play a significant role in meningitis development. Interestingly, another enzyme in the *dlt* operon, DltX, is also significantly increased in abundance in the MV-∆*iagB*. We previously observed that GBS∆*iagB* possesses more LTA compared to GBS WT strains (13), and the increased abundance of *dlt* operon enzymes in the MV-∆*iagB* may be a result of the increased LTA molecules in the bacterial cell.

To further understand the mechanisms by which GBS MVs induce inflammatory signaling in bECs, we treated MVs with a proteinase to digest surface-exposed proteins and observed significant decreases in the production of CXCL1 and IL-6, which is consistent with the activation of the NLRP3 inflammasome in macrophages (22). We also assessed the involvement of TLR2 as this is a major host cell receptor activated by the lipoproteins of bacteria and MVs from different species (17, 38). Using a small molecule inhibitor, which prevents TLR2/1 and TLR2/6 signaling in human cells (40), to block TLR2 signaling in bECs, we observed a significant decrease in CXCL-1 production and reduced levels of IL-6, further suggesting that the bECs’ recognition of GBS MVs is TLR2-dependent. While more investigation into the exact host cell signaling pathways that are activated by GBS MVs is needed, the identification of TLR2 in bECs and the NLRP3 inflammasome in macrophages may further help elucidate mechanisms by which GBS causes barrier dysfunction during neonatal meningitis. To confirm the inflammatory potential of MVs *in vivo*, we infected neonatal mice directly into the CSF via an ICV injection and observed that GBS MVs resulted in increased inflammation within the leptomeninges and brain, characterized by the upregulation of CXCL1, IL-6, TNFα, and IL-1β compared to liposome controls. These results are consistent with the inflammatory profile observed in the choriodecidua and macrophages in response to GBS MVs (22, 24) and during clinical GBS meningitis and suggest that MVs may contribute to the cytokine storm that drives neuroinflammation and subsequent brain injury. MVs from many bacterial species are known to induce inflammation and modulate host cell signaling (16, 17, 19, 37, 38) through host cell recognition of the MV surface proteins and LTA, both of which are potent TLR2 ligands (41, 42).

A major hurdle in the MV field is the biological implication of MV biogenesis in the host environment. Host cells produce extracellular vesicles, which are difficult to distinguish from bacterial MVs, making it challenging to determine the abundance of bacterial MV production during systemic infection in murine infections. Additionally, the cargo that is packaged into MVs is currently unknown under host conditions, although *in vitro* studies suggest that the protein and RNA composition will vary depending on the immune cell or host cell with which GBS interacts (16, 25, 32), and further investigation is ongoing to address these questions. During dissemination and systemic spread of GBS in the neonate, GBS will interact with innate immune cells, specifically with neutrophils, as they are the primary responders to invading pathogens in the bloodstream (43, 44). A previous study observed that GBS MVs will protect against reactive oxygen species produced by neutrophils and induce cytotoxicity (24). The fact that we identified NucA in GBS MVs suggests that they may play important roles during interactions with neutrophils and may potentially degrade the neutrophil extracellular traps (NETs). However, further investigation into the impact of GBS MVs on phagocytic uptake, NETosis, neutrophil inflammation, and cell death is warranted, as these mechanisms could have major impacts for GBS survival in the neonate.

In conclusion, our data indicate that while GBS membrane lipids are not essential for MV biogenesis, they do influence protein cargo. Despite changes in cargo, GBS MVs remain highly proinflammatory and can directly contribute to the pathogenesis of neonatal meningitis by triggering innate immune responses in bECs and the leptomeninges, potentially priming or exacerbating barrier dysfunction during infection. Additionally, how glycolipids impact MV direct interactions with endothelial, epithelial, or immune cells warrants further investigation. GBS MVs offer multiple potential uses: first, as diagnostic tools for GBS colonization and disease of neonates, and second, they may aid vaccine development due to their proinflammatory nature. Thus, future studies are required to identify the specific protein components within GBS MVs that drive these proinflammatory responses.

## MATERIALS AND METHODS

### Bacterial strains and growth conditions

GBS strains COH1 WT (28), COH1∆*iagB* (13), COH1∆*iagA* (15), and COH1∆*mprF* (14) were grown statically at 37°C in Todd-Hewitt Broth.

### Membrane vesicle isolation

MVs were isolated using ExoQuick-TC buffer (System Biosciences, Palo Alto, CA) as previously described (32, 38) and per the manufacturer’s protocol. Briefly, bacterial cultures grown for 18 h were pelleted at 3,900 × *g* in an Eppendorf 5810R centrifuge at 4°C for 15 min. Culture supernatants were sterile-filtered using a 0.22 µm filter before concentration using a 100 kDa molecular weight cutoff (Thermo Scientific) at 3,900 × *g* at 4°C. Buffer was added to the concentrated supernatants and incubated on ice for approximately 3 h before pelleting at 1,750 × *g* in an Eppendorf 5424 centrifuge for 30 min. Excess supernatant was removed, and MV pellets were stored at −80°C until use. MVs were resuspended in PBS (Gibco) or EndoGRO-MV (Millipore), depending on the assay.

### MV nanoparticle tracking

MVs were isolated in biological triplicate as described, and NanoSight measurements were performed by System Biosciences (Palo Alto, CA). Briefly, MVs were isolated as described above and resuspended in 200 µL of 0.02 µm filtered PBS. Particles were analyzed on a LM10 NanoSight (Malvern Panalytical) instrument in technical triplicate per sample.

### Lipidomic analyses

Acidic Bligh-Dyer extractions and liquid chromatography/electrospray ionization mass spectrometry (LC-MS/MS) were performed as previously described (14, 29, 45, 46). See Text S1 for more detail.

### Membrane vesicle proteomics

MVs were isolated as described, and proteomics were performed by System Biosciences (Palo Alto, CA) in biological triplicate. See Text S1 for more detail. Subcellular localization was identified by running GBS COH1 protein-coding sequences through pSortDB4 (47). Proteins designated as unknown were processed through CELLO (48) to further identify putative localization. Proteins with >5 peptides and present in all three biological replicates were included in analyses in DEP2 (30).

### Bacterial cell surface proteomics

In total, 12 mL of 18 h cell cultures were pelleted and washed once in protoplast buffer (49) (20% sucrose, 20 mM Tris-HCl [pH 7.0], and 10 mM MgCl_2_) supplemented with 50 µL of 2.5 KU mutanolysin (Sigma) and 25 µL proteinase inhibitor (Millipore). Cells were incubated at 37°C with rotation for approximately 2 h 30 min before protoplasts were pelleted at 15,000 × *g* in an Eppendorf 5424 tabletop centrifuge for 3 min. The supernatant was sterile-filtered using a SpinX 0.22 µm filter (Costar) for 1 min at 13,000 × *g* and kept on ice. Cell membranes were isolated as previously described (50). Briefly, protoplasts were resuspended in Tris buffer (pH 8, 20 mM) and sonicated for three rounds of 5 min cycling every 5 s (QSonica) on ice. Debris was pelleted at 15,000 × *g* for 5 min before ultracentrifugation at 110,000 × *g* at 4°C for 2 h using a Beckman Coulter Optima TLX ultracentrifuge. Cell surface preps and isolated cell membranes were combined, and proteins were precipitated using 10% Trichloroacetic acid (MP Biomedicals) for 18 h at 4°C and pelleted at 15,000 × *g* for 15 min for removal of buffer and snap frozen and stored at −80°C. Proteomic analysis was performed by Creative Proteomics (Shirly, NY). See Text S1 for more detail. Proteins present in all three biological replicates were included in analyses in DEP2 (30).

### RNA sequencing and data analysis

RNA was isolated in biological triplicate as previously described (32) using the miRNeasy micro kit (Qiagen) and sequenced by SeqCenter (Pittsburgh, PA) to obtain 12 M reads per sample. RNA reads were aligned to the GBS COH1 genome (HG939456) and counted using EDGE-Pro (51) with default settings. Gene counts were imported into RStudio for analysis. Initially, rRNA and tRNA reads were removed, and coding genes were filtered to have greater than three reads in at least two biological replicates to be kept for analysis; a pseudo count of 1 was used. Differential expression was performed using DESeq2 (52), and graphs were generated using ggplot.

### hCMEC cell inflammation and cytotoxicity assays

hCMEC/D3 (obtained from Millipore) were grown in EndoGRO-MV complete medium (Millipore, SCME004) supplemented with 5% fetal bovine serum (FBS) and 1 ng/mL fibroblast growth factor-2 (FGF-2; Millipore). Cells were grown in tissue culture-treated 24-well plates coated with 1% rat tail collagen (Corning) and 5% CO_2_ at 37°C to confluency. Before infection, cells were serum-starved for approximately 2 h by gently washing cells once with PBS (Gibco) before fresh, serum-free, EndoGRO-MV basal medium was added to the wells and incubated at 37˚C and 5% CO_2_. Immediately before infection, the medium was removed, and 200 µL of fresh serum-free medium was added, followed by 100 µL of MV’s normalized to indicated protein concentrations in serum-free basal medium. Plates were centrifuged for 5 min at 811 × *g* in an Eppendorf 5810R centrifuge. Proteinase K treatment was performed as previously described (39). MVs were normalized in 100 µL EndoGRO-MV basal medium and treated with 5 µg proteinase K (Roche) or sterile water vehicle control and incubated at 37˚C for 1 h before the addition of phenol-methysulfonyl fluoride (Amresco) to a final concentration of 0.1 mM to stop the activity of proteinase K before the addition to hCMECs; 100 µM of TLR2 inhibitor (TLR2-in-C29, Sigma) was incubated with cells ~2 h before the addition of MVs and remained present during infection. All assays were performed in biological triplicate with two technical replicates. For cytotoxicity assays, confluent hCMEC monolayers were infected with 70 µg of MVs for 24 h in complete medium described above, and lactate dehydrogenase (LDH) release was measured using the CyQUANT LDH release assay (Thermo Fisher) per the manufacturer’s protocol. All assays were performed in biological triplicate with at least two technical replicates.

### Enzyme-linked immunosorbent assay

Human CXCL-1 and IL-6 from hCMEC cell supernatants were detected by ELISA according to the manufacturer’s instructions (R&D Systems).

### Neonatal murine infection and leptomeningeal collection

Intracerebroventricular injection (ICV) of P1 C57Bl/6J mice were performed as previously described (33, 34). Briefly, neonatal mice were cryo-anesthetized by wrapping in a nitrile glove and covered in ice for approximately 5 min; 3 µL containing 9 µg of MVs or liposome (Encaposome) control was loaded onto a sterile Hamilton syringe (Hamilton) with a 32 g needle. Needles were held perpendicular to the skull and inserted 3 mm at the injection site approximately two-fifths of the distance between the lambda and the right eye. Following the injection, mice were placed on a heating pad until active and returned to their original cage. After 24 h, mice were euthanized via exposure to isoflurane, perfused with PBS, and whole brains were harvested. Leptomeninges were carefully removed from the brain, and both tissue types were frozen on dry ice and stored at −80°C until use.

### Multiplex cytokine assay

Multiplex cytokine array was performed by the Human Immune Monitoring Resource at the University of Colorado Anschutz. Briefly, leptomeninges and brain tissue were homogenized using Lysis Buffer containing 2× Proteinase Inhibitor cocktail per manufacture protocol (Meso Scale Diagnostics) and stored at −80˚C until use. Samples were thawed and spun at 2,000 × *g* for 3 min. Pre-coated V-PLEX plates were washed using an automated plate washer (BioTek ELX5012), 50 µL of calibrators or diluted plasma samples were added, and plates were incubated for 2 h at room temperature on a Compact Digital Microplate shaker (Thermo Fisher) at 600 rpm. Plates were washed and 25 µL of the diluted detection antibodies was added and incubated for 2 h at room temperature. After washing, 2× read buffer (MesoScale Discovery) was added, and plates were immediately read on a MesoQuickPlex SQ120 electrochemiluminescent plate reader. Data were analyzed using Workbench software (MesoScale Discovery).

### Statistical analyses

All statistics were performed using GraphPad Prism software V11. All relevant statistical analyses are indicated in figure legends.

## Data Availability

RNA-seq reads are available under NCBI SRA accession number PRJNA1441633. The MV proteomic data have been deposited to the ProteomeXchange Consortium via the PRIDE (53) partner repository with the data set identifier PXD076412.

## SUPPLEMENTAL MATERIAL

Supplemental material iai.00231-26-s0001.pdf Fig. S1 to S4.

Table S1 iai.00231-26-s0002.xlsx All proteins detected in GBS MVs.

Table S2 iai.00231-26-s0003.xlsx Differential abundance of cell surface proteins in MV-∆iagB vs MV-COH1.

Table S3 iai.00231-26-s0004.xlsx Differential abundance of cell surface proteins in MV-∆mprF vs MV-COH1.

Table S4 iai.00231-26-s0005.xlsx Cell surface protein identification in parent GBS cells.

Table S5 iai.00231-26-s0006.xlsx Differential abundance of cell surface proteins in parent GBS cells.

ASM does not own the copyrights to Supplemental Material that may be linked to, or accessed through, an article. The authors have granted ASM a non-exclusive, world-wide license to publish the Supplemental Material files. Please contact the corresponding author directly for reuse.
