# Supplementary material for: Group B streptococcal membrane vesicles induce proinflammatory responses in neonatal meninges
Source: Infect Immun. 2026 Jun 22;94(7):e00231-26. doi: 10.1128/iai.00231-26 (PMC13367065; doi:10.1128/iai.00231-26)
Supplement: Supplemental material — Fig. S1 to S4. [file iai.00231-26-s0001.pdf]

Supplemental Tables, Figures, and Text

Group B Streptococcal Membrane Vesicles Induce Proinflammatory Responses in Neonatal Meninges

Luke R. Joyce[1*], Amanda Brady[1], Sol Kim[2], Priya M Christensen[3], Kelli L Palmer[3], Ziqiang Guan[4], Julie A Siegenthaler[2], Kelly S. Doran[1*]

Table S1: All proteins detected in GBS MVs

Table S2: Differential abundance of cell surface proteins in MV-Δ*iagB* vs MV-COH1

Table S3: Differential abundance of cell surface proteins in MV-Δ*mprF* vs MV-COH1

Table S4: Cell surface protein identification in parent GBS cells

Table S5: Differential abundance of cell surface proteins in parent GBS cells

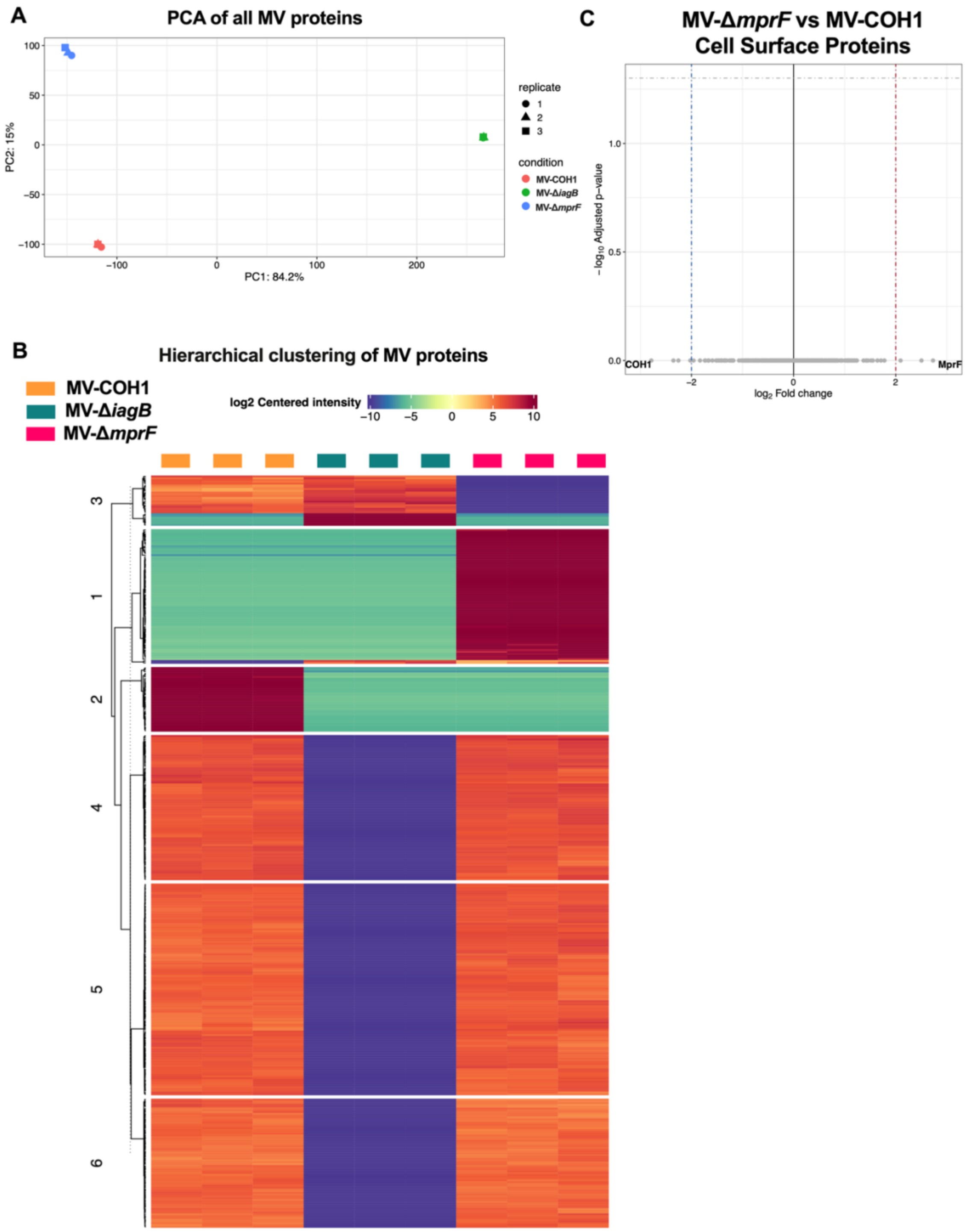

Figure S1. MV proteome alterations. A) Principal component analysis (PCA) of MV proteins, B) hierarchical clustering of proteins detected in MVs and C) differential abundance of MV-Δ*mprF* cell surface proteins compared to MV-COH1. Proteomics performed in biological triplicate.

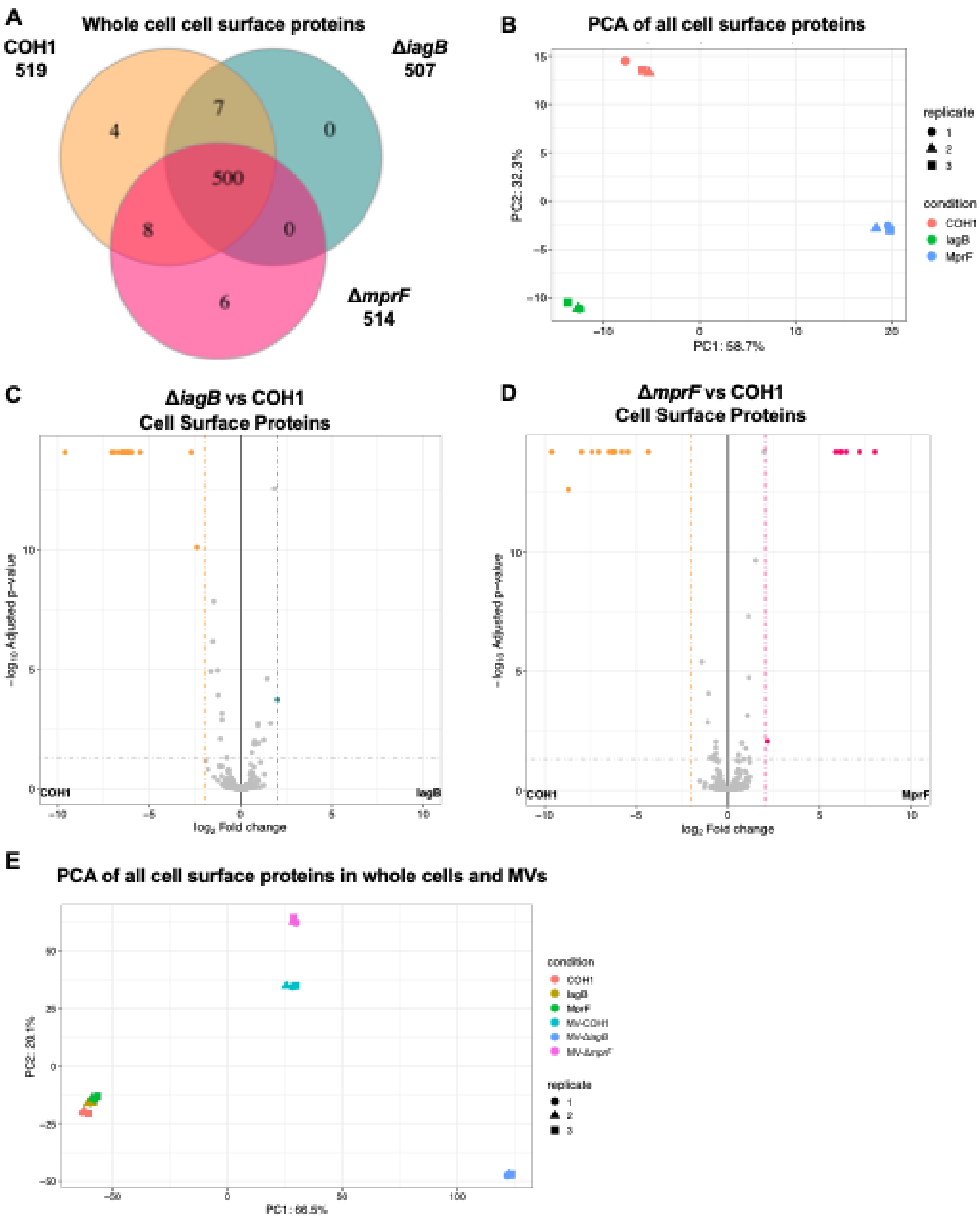

Figure S2. GBS bacterial cell surface proteome analysis. A) Total number of cell surface proteomes identified in live GBS, B) PCA analysis of cell surface proteins, and differential abundance of cell surface proteins of C) Δ*iagB* and D) Δ*mprF* compared to COH1 WT. E) PCA analysis of cell surface proteins from live stationary phase whole cells and associated MVs.

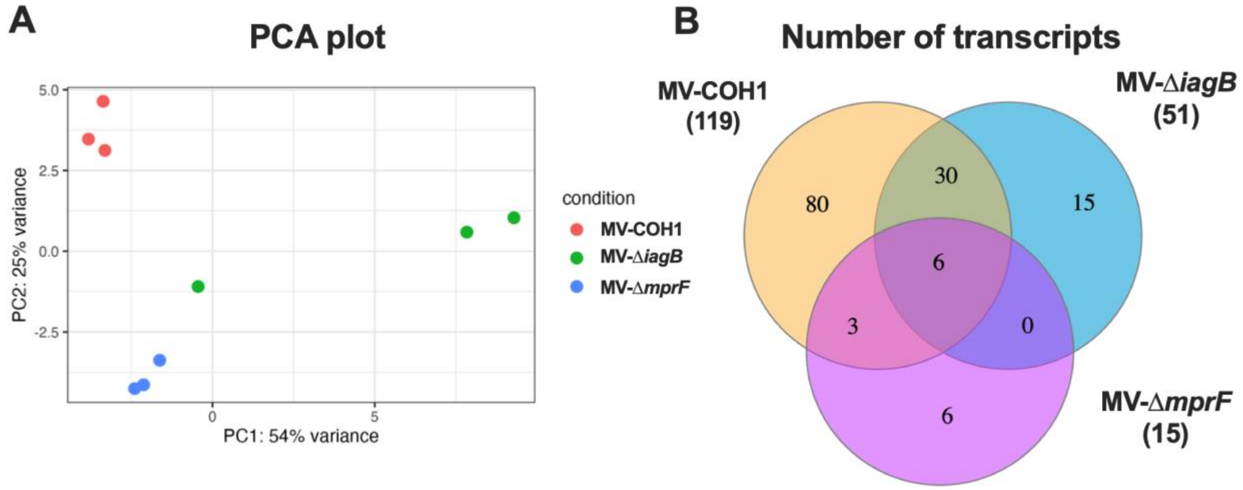

C

Signficantly altered transcripts compared to WT

MV-Δ*iagB* Gene	L2FC	pAdj	MV-Δ*mprF* Gene	L2FC	pAdj
GBSCOH1_1776	-4.48	5.23E-08	GBSCOH1_1777	6.51	1.14E-07
GBSCOH1_0416	5.40	0.0002	GBSCOH1_1132	4.28	0.0112
GBSCOH1_1246	4.72	0.0017	GBSCOH1_0328	4.28	0.0112
GBSCOH1_0853	4.55	0.0024	GBSCOH1_0288	4.28	0.0112
GBSCOH1_0905	4.46	0.0029	GBSCOH1_1280	4.15	0.0186
GBSCOH1_0829	4.36	0.0038	GBSCOH1_0275	4.01	0.0220
GBSCOH1_0008	4.26	0.0045			
GBSCOH1_0945	4.26	0.0045			
GBSCOH1_0383	4.15	0.0061			
GBSCOH1_0572	4.02	0.0072			
GBSCOH1_1098	4.02	0.0072			
GBSCOH1_1237	4.02	0.0072			
GBSCOH1_0666	3.89	0.0103			
GBSCOH1_1222	2.74	0.0103			
GBSCOH1_0132	3.74	0.0140			
GBSCOH1_0510	3.74	0.0140			
GBSCOH1_0879	3.74	0.0140			
GBSCOH1_0837	2.35	0.0404			
GBSCOH1_1327	2.08	0.0404			

Figure S3. RNA content of MVs. A) PCA plot of RNA from each MV. B) Venn-diagram indicating the number of unique and shared transcripts identified in MVs. C) Significantly altered transcripts identified by DESeq2 in MV-Δ*iagB* and MV-Δ*mprF* compared to MV-COH1. Biological triplicate performed.

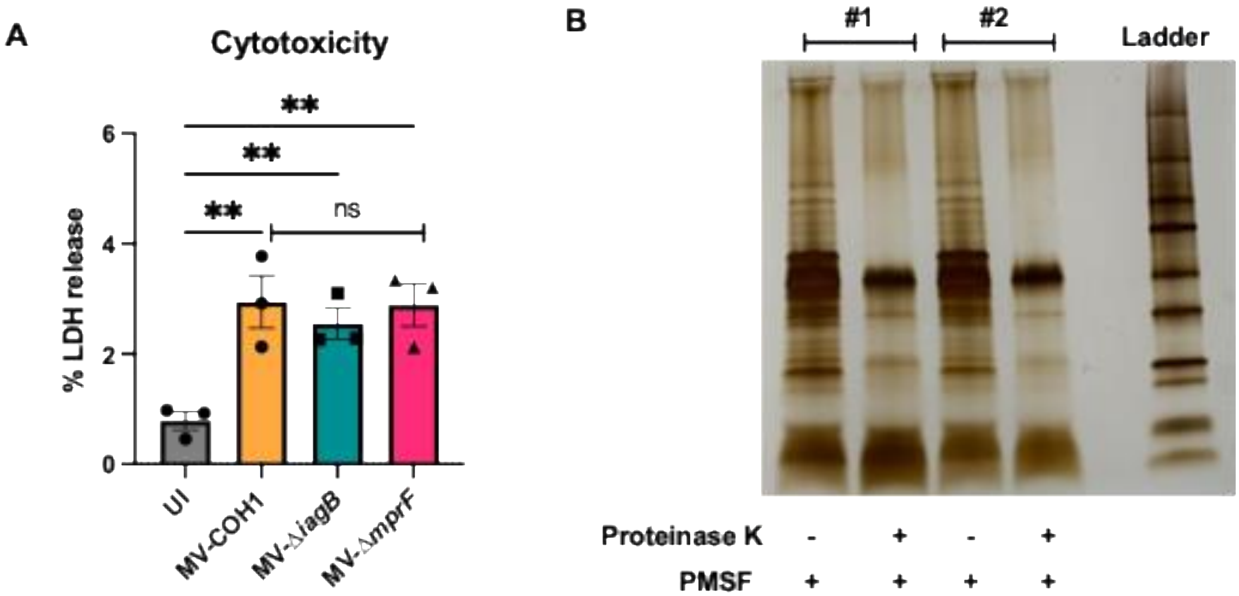

Figure S4. hCMEC LDH release and silver stain gel of MV-COH1 digested by proteinase K. A) Percent lactate dehydrogenase (LDH) release by hCMEC's incubated with 70 µg of MV-COH1, MV-Δ*iagB*, and MV-Δ*mprF* for 24 h indicates low levels of cytotoxicity. Three biological replicates with at least 2 technical replicates each, median and SEM. One-way ANOVA with Fisher's LSD test. **p<.01, ns; not significant. B) Confirmation of protein digestion by proteinase K, two biological MV preparations are indicated.

Supplemental Text S1.

Acidic Bligh-Dyer extractions

Centrifugation was performed using a Sorvall RC6+ centrifuge. MVs were stored at -80°C until acidic Bligh-Dyer lipid extractions were performed as described (1). Briefly, MVs were resuspended in 1X PBS (Sigma-Aldrich) and transferred to Coring Pyrex glass tubes with PTFR-lined caps (VWR), followed by 1:2 vol:vol chloroform:methanol addition. Single phase extractions were vortexed periodically and incubated at room temperature for 15 minutes before 500 x *g* centrifugation for 10 min. A two-phase Bligh-Dyer was achieved by addition of 100 µL 37% HCl, 1 mL $CHCl_3$, and 900 µl of 1X PBS, which was then vortexed and centrifuged for 5 min at 500 x *g.* The lower phase was removed to a new tube and dried under nitrogen before being stored at -80°C prior to lipidomic analysis.

Liquid Chromatography/Electrospray Ionization Mass Spectrometry

Normal-phase LC was performed on an Agilent 1200 quaternary LC system equipped with an Ascentis silica HPLC column (5 µm, 25 cm × 2.1 mm; Sigma-Aldrich) as described previously (2, 3). Briefly, mobile phase A consisted of chloroform–methanol–aqueous ammonium hydroxide (800:195:5, vol/vol), mobile phase B consisted of chloroform–methanol–water–aqueous ammonium hydroxide (600:340:50:5, vol/vol), and mobile phase C consisted of chloroform–methanol–water–aqueous ammonium hydroxide (450:450:95:5, vol/vol). The elution program was as follows: 100% mobile phase A was held isocratically for 2 min, then linearly increased to 100% mobile phase B over 14 min, and held at 100% mobile phase B for 11 min. The gradient was then changed to 100%

mobile phase C over 3 min and held for 3 min, before returning to 100% mobile phase A over 0.5 min and holding for 5 min. The LC eluent (total flow rate of 300 μL/min) was introduced into the HESI source of an Orbitrap Exploris™ 120 mass spectrometer (Thermo Fisher Scientific Inc., Waltham, MA). Instrument settings for negative-ion ESI and MS/MS analysis of lipid species were as follows: spray voltage, −2,800 V; sheath gas (arb), 40; auxiliary gas (arb), 15; sweep gas (arb), 5; vaporization temperature, 280 °C; ion transfer tube temperature, 320 °C; and collision gas (nitrogen) pressure, 1 mTorr. For untargeted data-dependent acquisition, the instrument was operated with the following MS1 settings: automatic gain control (AGC), standard; resolution, 30,000; maximum injection time, 50 ms; and scan range, m/z 100–2000. Tandem MS data were acquired in a data-dependent mode on the four most abundant precursor ions, with a 2 m/z isolation window, a minimum AGC target of 5×10^5, and dynamic exclusion set to 20 s. Data analysis was performed using the FreeStyle software (Thermo Fisher Scientific Inc).

MV proteomics

Samples were concentrated by ultrafiltration and run into SDS-PAGE as gel plugs. Each gel plug was subjected to reduction with 10mM DTT for 30 min at 60˚C, alkylation with 20mM iodoacetamide for 45min at room temperature in the dark and digestion with trypsin (Thermo Scientific), and incubated over-night at 37°C. Peptides were extracted twice with 5% formic acid, 60% acetonitrile and dried under vacuum. Samples were analyzed by liquid chromatography-tandem mass spectrometry (LC-MS/MS) using Nano LC-MS/MS (Dionex Ultimate 3000 RLSCnano System, Thermofisher) interfaced with Eclipse (Thermofisher). Samples were loaded on to a fused silica trap column Acclaim PepMap

100, 75umx2cm (ThermoFisher). After washing for 5 min at 5 µl/min with 0.1% TFA, the trap column was brought in-line with an analytical column (Nanoease MZ peptide BEH C18, 130A, 1.7um, 75umx250mm, Waters) for LC-MS/MS. Peptides were fractionated at 300 nL/min using a segmented linear gradient 4-15% B in 30min (where A: 0.2% formic acid, and B: 0.16% formic acid, 80% acetonitrile), 15-25%B in 40min, 25-50%B in 44min, and 50-90%B in 11min. Solution B then returns at 4% for 5 minutes for the next run. DIA (Data independent acquisition) workflow was used to analyze the eluted peptides. MS scan range were set at 400-1200, resolution 12,000 with AGC set at 3E6 and ion time set as auto. 8 m/z window were set to sequentially isolate (AGC 4E5 and ion time set at auto) and fragment the ions in C-trap with relative collision energy of 30. The MSMS were recorded with Resolution of 30,000. Raw data were analyzed with predicted library from specified database and for library-free search using DIA NN 1.8.1 (4) with recommended setting.

Bacterial cell surface proteomics.

Sample Preparation

Samples were added urea to a final concentration to 8 M, and appropriate 1x protease inhibitor cocktail was also added to inhibit protease activity. The mixture was treated by ultrasonic for 5 min and was allowed to settle at 4℃ for 30 min. After centrifugation at 16,000 x *g* for 20 min at 4˚C, the supernatant was collected. Then the concentration of protein supernatant was determined by BCA Protein Assay Kit (ThermoScientific) per manufacturer protocol.

Protein digestion

Sample preparation contains the process of protein denaturation, reduction, alkylation as well as the tryptic digestion and peptide cleanup. Commercial iST Sample Preparation kit (PreOmics, Germany) was used according to protocols provided. Briefly, the protein samples were heated at 95°C for 10 min at 1000 rpm with agitation. After cooling to room temperature, trypsin digestion buffer was added, and the sample incubated at 37˚C for 2 h at 500 rpm with shaking. The digestion process was stopped with a stop buffer. Sample clean-up and desalting was carried out in the iST cartridge using the recommended wash buffers. Peptides were eluted with elution buffer (2 × 100 μL) and then dried in a speed vacuum concentrator.

DIA data collection

The nanoElute 2 (Bruker Daltonik, Bremen, Germany) liquid chromatography system was connected to the timsTOF Pro 2, an ion-mobility spectrometry quadrupole time of flight mass spectrometer (Bruker Daltonik, Bremen, Germany). Samples were reconstituted in 0.1% FA and 200 ng peptide was separated by AUR3-15075C18 column (15 cm length, 75 µm i.d, 1.7 µm particle size, 120 Å pore size) with a 60 min gradient starting at 2.2% buffer B (80% ACN with 0.1% FA) followed by a stepwise increase to 28% in 46 min, 38% in 8min ,90% in 3 min and stayed there for 3 min. The column flow rate was maintained at 400 nL/min with the column temperature of 50°C. DIA data was acquired in the diaPASEF mode. We defined 22 × 40 Th precursor isolation windows from m/z 349 to 1229. To adapt the MS1 cycle time, we set the repetitions to variable steps (2-5) in the 13-scan diaPASEF scheme in our experiment. During PASEF MSMS scanning, the

collision energy was ramped linearly as a function of the mobility from 59 eV at 1/K0 = 1.6 Vs/cm2 to 20 eV at 1/K0 = 0.6 Vs/cm^2.

DIA database search

Raw Data of DIA were processed and analyzed by Spectronaut 19 (Biognosys AG, Switzerland) with default settings using the protein sequencing from GBS COH1 whole genome accession number HG939456. Trypsin was the digestion enzyme and specific was the digest type. Carbamidomethyl on cysteine was specified as the fixed modification. Oxidation on methionine, Acetyl on protein N-term were specified as the variable modifications. Retention time prediction type was set to dynamic iRT. Data extraction was determined by Spectronaut based on the extensive mass calibration. Spectronaut will determine the ideal extraction window dynamically depending on iRT calibration and gradient stability. Qvalue (FDR) cutoff on precursor level was 1%, peptide level was 1% and protein level was 1%. Decoy generation was set to mutated which similar to scrambled but will only apply a random number of AA position swamps (min = 2, max = length/2). Normalization strategy was set to Local normalization. Peptides which passed the 1% Qvalue cutoff were used to calculate the major group quantities with MaxLFQ method.

References

1. Joyce LR, Guan Z, Palmer KL. 2019. Phosphatidylcholine Biosynthesis in Mitis Group Streptococci via Host Metabolite Scavenging. *J Bacteriol* 201:e00495-19.
2. Tan BK, Bogdanov M, Zhao J, Dowhan W, Raetz CR, Guan Z. 2012. Discovery of a cardiolipin synthase utilizing phosphatidylethanolamine and phosphatidylglycerol as substrates. *Proc Natl Acad Sci U S A* 109:16504-9.

3. Li C, Tan BK, Zhao J, Guan Z. 2016. In Vivo and in Vitro Synthesis of Phosphatidylglycerol by an Escherichia coli Cardiolipin Synthase. *J Biol Chem* 291:25144-25153.
4. Demichev V, Messner CB, Vernardis SI, Lilley KS, Ralser M. 2020. DIA-NN: neural networks and interference correction enable deep proteome coverage in high throughput. *Nat Methods* 17:41-44.
